# The D2.B10-*Dmd*^*mdx*^/J Mouse Model of Duchenne Muscular Dystrophy Exhibits a Severe Mitochondrial Deficiency Not Observed in the C57BL/10ScSn-*Dmd*^*mdx*^/J Mouse

**DOI:** 10.1016/j.ajpath.2025.09.005

**Published:** 2025-09-30

**Authors:** Jennifer A. Tinklenberg, Jessica Sutton, Rebecca A. Slick, Hui Meng, Margaret Haberman, Mariah J. Prom, Margaret J. Beatka, Tatyana A. Vetter, Audrey L. Daugherty, Christina A. Pacak, J. Patrick Gonzalez, Michael W. Lawlor

**Affiliations:** ∗ Division of Pediatric Pathology, Department of Pathology and Laboratory Medicine and Neuroscience Research Center, Medical College of Wisconsin, Milwaukee, Wisconsin; † Department of Physiology, Medical College of Wisconsin, Milwaukee, Wisconsin; ‡ Clinical and Translational Science Institute, Medical College of Wisconsin, Milwaukee, Wisconsin; § Greg Marzolf Jr. Muscular Dystrophy Center and Department of Neurology, University of Minnesota, Minneapolis, Minnesota; ¶ Diverge Translational Science Laboratory, Milwaukee, Wisconsin; ‖ Solid Biosciences, Charlestown, Massachusetts

## Abstract

Duchenne muscular dystrophy (DMD) is caused by mutations in the *DMD* gene, resulting in dystrophin deficiency in skeletal/cardiac muscle and progressive loss of function. Although the genetic causes of DMD have been thoroughly investigated, the energetic consequences have not been well examined across animal models. Previously, the laboratory examined mitochondrial function across nemaline myopathy mouse models of varying disease severity; here, mitochondrial phenotypes in DMD are assessed through the comparison of the milder C57BL/10ScSn-*Dmd*^*mdx*^/J (B10-*mdx*) and the more severe D2.B10-*Dmd*^*mdx*^/J mouse (D2-*mdx*) mouse models. D2-*mdx* exhibit a significant decrease in mitochondrial respiration, undetectable ATP concentrations, increased mitochondrial membrane potential, and alterations in electron transport chain enzyme activities. In contrast, B10-*mdx* show only mild mitochondrial phenotypes, including decreased ATP content. The D2-*mdx* mouse has genetic modifiers, including latent transforming growth factor-β–binding protein 4 (LTBP4) and annexin A6, that have been shown to alter DMD severity in humans. However, these modifiers did not account for mitochondrial differences seen in *mdx* mice. Both models were treated with a microdystrophin adeno-associated virus gene therapy to assess whether dystrophin restoration rescued mitochondrial phenotypes. Gene therapy attenuated the ATP deficiency in the B10-*mdx* mice, but only improved mitochondrial membrane potentials in D2-*mdx* mice. The exact cause of the D2-*mdx* mitochondrial phenotypes remains unknown, but secondary disease processes that affect mitochondrial phenotypes should be taken into consideration when choosing an animal model for DMD studies.

Duchenne muscular dystrophy (DMD) is a progressive X-linked degenerative muscle disorder caused by mutations in the *DMD* gene, which encodes the muscle protein dystrophin.^1^ A deficiency in dystrophin results in an unstable interaction between dystrophin, F-actin, and the extracellular matrix, therefore weakening the sarcolemma (previously reviewed^1^). Patients with DMD have impaired respiratory and cardiac functions, orthopedic complications, including contractures and scoliosis, slowed gastrointestinal emptying, and cognitive impairment or behavioral problems.^1^ Subsequently, management of DMD symptoms requires a multidisciplinary approach because of the wide variety of symptoms. Treatments for DMD include supportive care, glucocorticoid treatments,^1^ and recently approved and experimental treatments that include gene therapies to repair or provide a functional copy of *DMD*^1^ in the form of microdystrophin (previously reviewed^2^). Multiple microdystrophin constructs have been produced that include critical functional dystrophin domains, thus allowing them to fit inside the adeno-associated virus (AAV) vectors used for gene delivery.^2^ Treatment of DMD with microdystrophin AAVs has shown favorable results for DMD pathology in mice and dogs, including improved pathology, limb muscle function, gait, and clinical score.^1^, ^2^, ^3^, ^4^

Two of the most commonly used mouse models for DMD preclinical studies are the C57BL/10ScSn-*Dmd*^*mdx*^/J (B10-*mdx*) and D2.B10-*Dmd*^*mdx*^/J (D2-*mdx*) mice. The B10-*mdx* mouse model was first identified as a spontaneous mutation in an inbred C57Bl/10 colony and described in 1984.^5^ Since then, it has become the most widely used mouse model for DMD. These mice have a milder phenotype than most patients and, unlike humans with DMD, they have minimal fatty deposits or fibrosis in the muscle tissue.^6^ The second mouse model, the D2-*mdx*, was developed by backcrossing the B10-*mdx* mouse onto a DBA/2J background to generate a mouse with a more similar phenotype to human patients.^7^ The DBA/2J background introduces several genetic modifiers that have been shown to influence DMD severity, including changes to *Ltbp4*, which affects transforming growth factor (TGF)-β signaling,^8^ *Anxa6*, which alters satellite cell regeneration,^9^ and *Dyscalc1*, which is believed to cause calcifications in the skeletal muscle and heart.^10^

Previously published studies described mitochondrial alterations in three mouse models of nemaline myopathy (NM) caused by mutations in the skeletal muscle actin (*Acta1*) and nebulin (*Neb*) genes.^11^, ^12^, ^13^, ^14^, ^15^, ^16^, ^17^ On the basis of protein expression data that demonstrated abnormalities related to metabolism in these NM mouse models, mitochondrial function was evaluated using commercially available assays.^14^^,^^15^ These assessments demonstrated that the degree of mitochondrial dysfunction correlated with the severity of myopathy in each mouse model.^14^^,^^15^ Overall, the assessments proved to be a valuable screen to identify (presumably secondary) mitochondrial abnormalities in muscle disease.

Here, using similar techniques, the mitochondrial abnormalities between B10-*mdx* and D2-*mdx* mouse models of DMD and their respective wild-type (WT) control strains were assessed. Previous studies by other groups have identified a variety of mitochondrial and metabolic abnormalities in B10-*mdx* mice, both preceding and following the onset of muscle damage. These abnormalities included changes in mitochondrial morphology, electron transport chain (ETC) complex activity, respiratory function, ATP content, mitochondrial DNA (mtDNA) content, and gene expression.^16^, ^17^, ^18^, ^19^, ^20^, ^21^, ^22^, ^23^, ^24^, ^25^, ^26^, ^27^, ^28^, ^29^ However, changes in mitochondrial function have not been as thoroughly investigated in the D2-*mdx* mice, and have largely focused on heart function.^30^^,^^31^ These two models have yet to be assessed for mitochondrial function at a common time point to enable direct comparisons.

Once mitochondrial phenotypes were characterized between the B10-*mdx* and the D2-*mdx* mice, a pilot study was performed to determine how well systemic delivery of microdystrophin-5 (rAAV9-CK8-μDys5; SGT-001), courtesy of Solid Biosciences (Charlestown, MA), improved mitochondrial deficiencies in these models. SGT-001 is currently part of an ongoing human clinical trial (*https://clinicaltrials.gov/ct2/show/NCT03368742*, last accessed August 4, 2025) and has shown therapeutic benefit in DMD canine models.^32^ This transgene encodes for a dystrophin construct that maintains all the key components of full-size dystrophin while still remaining small enough to fit inside of an AAV.^33^ Treatment with microdystrophin has shown therapeutic benefits in dog models of DMD^34^^,^^35^ as well as the B10-*mdx*^36^^,^^37^ and D2-*mdx*^38^ mouse models in restoring muscle function. However, it is still unknown how functional dystrophin restoration affects secondary mechanisms of disease.

## Materials and Methods

### Live Animal Studies and Overall Study Design

All studies were performed with approval from the Institutional Animal Care and Use Committee at the Medical College of Wisconsin (Milwaukee, WI). Male mice were purchased from the Jackson Laboratory (Bar Harbor, ME) at 5 weeks of age and housed in groups of five mice per cage. Food and water were provided *ad libitum*, and mice were kept on a 16-hours light/8-hours dark cycle. Wild-type strains were the C57BL/10ScSnJ (B10-WT; strain number 000476) and DBA/2J (D2-WT; strain number 000671), which served as the control strains for the DMD mice, C57BL/10ScSn-Dmd^*mdx*^/J (B10-*mdx*; strain number 001801) and D2.B10-Dmd^*mdx*^/J (D2-*mdx*; strain number 013141), respectively. All mice were humanely euthanized at 10 weeks of life using CO_2_ with cervical dislocation, and muscles were removed, weighed, and processed according to procedures listed below in the Materials and Methods or frozen in liquid nitrogen-cooled isopentane^39^ for histologic evaluation.

For the first half of the study, overall mitochondrial function in the B10-WT, B10-*mdx*, D2-WT, and D2-*mdx* mice was evaluated by isolating mitochondria from muscle tissue and then evaluating mitochondrial respiration [respiratory control index (RCI)], mtDNA content, enzyme function, ATP and ADP quantification, and mitochondrial membrane potential (ΔΨ_m_). Histologic analysis was performed to visualize overall muscle morphology and mitochondrial location. Western blot analyses were performed to identify changes in protein expression in relation to mitochondrial dynamics.

Once a mitochondrial phenotype was established, *mdx* mice were treated with microdystrophin AAV to evaluate if dystrophin restoration improved mitochondrial function. B10-*mdx* mice were treated with 2.00 × 10^14^ vector genomes (vg)/kg of microdystrophin AAV, and D2-*mdx* mice were treated with 1.00 × 10^14^ vg/kg. After 1 month of treatment, mitochondrial evaluation was performed.

To reduce the number of mice needed for all studies, tissues and isolates from each mouse were used for as many functional assays as possible and are outlined below in the Materials and Methods. Exact sample numbers have been included in each graph.

### Mitochondrial Isolation

Mitochondria were isolated using a modified standard protocol, as previously described.^14^^,^^15^ First, each animal's quadriceps, gastrocnemius, hamstring, and triceps muscle was homogenized (*n* = 1) to achieve a mass <600 mg and centrifuged three times to isolate the mitochondrial pellet^14^ immediately after animal sacrifice. The mitochondrial pellet was resuspended in Isolation Buffer [200 mmol/L mannitol, 50 mmol/L sucrose, 5 mmol/L KH_2_PO_4_, 5 mmol/L 3-(N-morpholino) propanesulfonate, 1 mmol/L EGTA, and 0.1% bovine serum albumin, pH adjusted to 7.15 with KOH] and used for mitochondrial respirometry, ETC enzyme function, and ΔΨ_m_ immediately. Both WT and *mdx* samples were isolated using identical means and concurrently on any experimental day. These crude mitochondrial pellets were used to perform the following assays: RCI, ETC enzyme function assays, and the mitochondrial membrane potential.

### Respiratory Control Index

The mitochondrial RCI (also known as the respiratory control ratio) was performed to evaluate overall mitochondrial health. Mitochondrial isolates (22 μL at 12.5 mg/mL) from skeletal muscle were placed in a Clark Oxygen Electrode chamber (model S 200A; Strathkelvin Instruments, Glasgow, Scotland), as previously described.^14^^,^^15^ A glutamate/malate solution (final buffer concentration of 20 mmol/L) was added to the chamber with the mitochondria, followed by ADP, which the mitochondria use to consume oxygen as they convert ADP to ATP and water. The decrease in the amount of oxygen in the chamber over time (state 3) divided by the slope of the line after ADP consumption (state 4) was then used to calculate the RCI of the samples. WT samples were analyzed in the electrode first to ensure the machine was properly calibrated and the respiratory membrane was intact. After successful respiration with WT samples, *mdx* samples were analyzed and alternated with WT samples for each technical and biological replicate. If WT samples failed to respire with the addition of ADP, the respiratory membrane was replaced, and the electrode was recalibrated before running the sample again. If WT samples still failed to respire, all samples from that day's isolation were excluded from further testing. Alternating between WT and *mdx* tissue ensured proper electrode function.

### Relative mtDNA Content

Relative mtDNA content was assessed using real-time quantitative PCR on a QuantStudio 3 System (Applied Biosystems, Waltham, MA). Genomic DNA was isolated from the quadriceps of three mice from each genotype using the DNeasy Blood and Tissue Kit (Qiagen, Hilden, Germany). The concentrations (ng/μL) of genomic DNA and the purity ratios (A260/280 and A260/230) were assessed using a Nanodrop Spectrophotometer (Thermo Fisher, Waltham, MA). All purity ratios were within range, indicating no ethanol contamination or degradation of DNA quality. The assay was performed using 400 ng of genomic DNA for each mouse with TaqMan fast chemistries (Thermo Fisher). To evaluate mtDNA content, mouse TaqMan probes for mtDNA ND1 (Mm0422574_s1) and nuclear DNA Hbb-b1 (Mm01611268_g1) were used. Cycle threshold (C_T_) values were then used to determine a ΔC_T_ of mitochondrial content relevant to nuclear content, resulting in relative mtDNA content, as previously described.^40^

### ETC Enzyme Functional Assays

ETC enzyme activity assays were performed as previously described^14^^,^^15^ to assess the function of each enzyme in the ETC. WT and *mdx* mitochondria were examined concurrently, and all five enzymes were examined on the same day from each mitochondrial isolate. Samples were normalized so 5.5 mg/mL of protein was loaded into each sample well, and samples were run in duplicate. Mitochondrial isolates with sample concentrations of <5.5 mg/mL were excluded from the study as this was the minimum concentration needed to obtain functional results.^14^ Additionally, per the manufacturer's instructions, bovine heart mitochondria were used as a positive control for all assays. If the bovine heart mitochondria did not respond to the assay conditions, the samples from that day were excluded from the study.^14^^,^^15^ For all assays, WT and *mdx* samples were run on the same plate, and colorimetric change over time was assessed for each assay as a measure of enzyme activity. Details of each assay are summarized in Supplemental Table S1.^14^^,^^15^

### ATP and ADP Assays

Immediately after animal sacrifice, the amount of ATP and ADP was assessed to determine total content in skeletal muscle isolates. One triceps from each mouse was used for ATP (Abcam, Waltham, MA; ab83355) and one for ADP (Abcam; ab83359) content per the manufacturer's instructions and further detailed in Supplemental Table S1.^14^^,^^15^ Muscle was minced and homogenized in each assay's specific buffer and then centrifuged. The amount of supernatant used in the assay was adjusted so the samples' OD readings fell within the standard curve of the assay. Samples were then normalized to average protein concentration based on the μL of the sample added to the assay plate. Samples were analyzed immediately on preparation, and WT and *mdx* animals were run concurrently on the same plate. The ratio of [ATP]/[ADP] was also calculated for each sample.

### Mitochondrial Membrane Potential

Mitochondrial membrane potential was assessed using the Mitochondria Staining Kit (Sigma-Aldrich, St. Louis, MO; CS0760), per the manufacturer's instructions, on isolated mitochondria. Using JC-1 (5,5′,6,6′-tetrachloro1,1′,3,3′-tetraethylbenzimidazolocarbocyanine iodide), 5 μg of protein was loaded into black-walled 96-well plates for each sample. WT and *mdx* samples were evaluated on the same plate. The ratio of the red fluorescent JC-1 aggregates (excitation, 490 nm/emission, 590 nm) and green, fluorescent monomers (excitation, 485 nm/emission, 535 nm) was used to determine ΔΨ_m_ after a 20-minute incubation.

### Histologic Evaluation

Histologic evaluation was performed to assess muscle structure using hematoxylin and eosin (H&E) staining. Mitochondrial distribution was visualized using cytochrome *c* oxidase (COX) with succinate dehydrogenase (SDH) staining, which stains for complex IV and complex II activity, respectively. The quadriceps muscle of six mice from each genotype were cut at 8 μm. H&E and COX + SDH staining was performed on the tissue cross-sections using standard techniques.

Microdystrophin was assessed using immunofluorescence to show successful dosing with AAV and quantify microdystrophin expression. Quadriceps tissues were stained using laminin (Abcam; ab11576; 1:400) and MANDYS106 (Millipore Sigma, Burlington, MA; MABT827; 1:50) for human microdystrophin from the delivered AAV. Secondary antibodies included AF647 goat anti-rat IgG (Jackson ImmunoResearch, West Grove, PA; 112-605-167; 1:200) for laminin and goat anti-mouse IgG2a Secondary Antibody Alexa Fluor 546 (Invitrogen, Waltham, MA; A21133; 1:400) for microdystrophin. Whole-section images were captured on a motorized Nikon (Melville, NY) Ni-E microscope with a Hamamatsu (Puslinch, ON, Canada) ORCA Fusion camera and a Plan Apochromat λ 20× objective at a resolution of 0.32 μm/pixel.

**Supplemental Figure S1:**
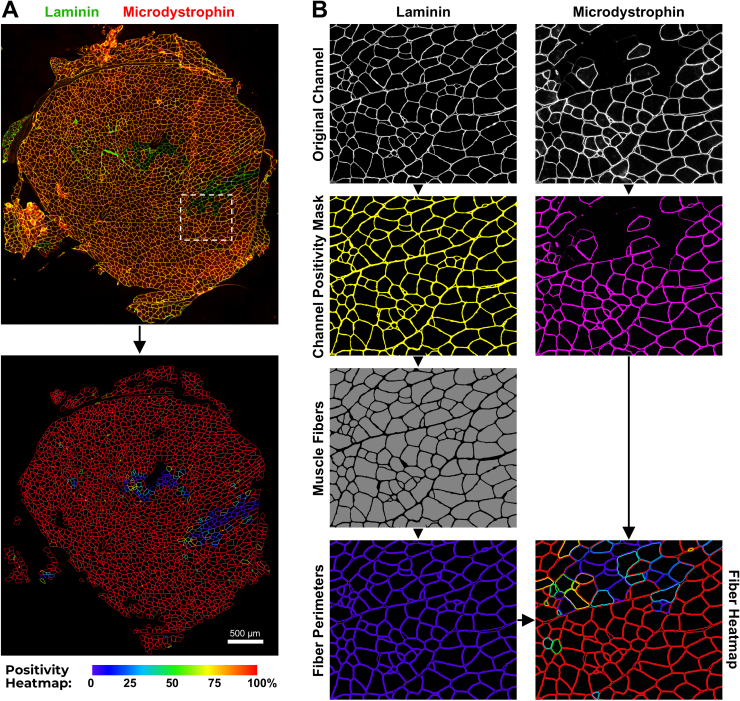
Microdystrophin image analysis. **A:** Representative image of a whole section stained for microdystrophin and laminin with the corresponding microdystrophin quantification heat map from an adeno-associated virus–treated B10-*mdx* mouse. The heat map scale reflects the percentage microdystrophin-positive perimeter of each muscle fiber, and fibers with at least 30% positive perimeter are considered overall positive. The **white boxed area** is the zoomed area indicated in **B**. **B:** The microdystrophin quantification workflow automatically segments laminin- and microdystrophin-positive pixels, derives muscle fibers automatically from the laminin signal, and determines the percentage microdystrophin-positive perimeter from the laminin and microdystrophin binary layers within a 3-μm perimeter ring around each muscle fiber. Scale bar = 500 μm (**A**).

Microdystrophin immunofluorescence was quantified using NIS-Elements AR software version 6.1 (Nikon) with the General Analysis 3 software module and a custom analysis workflow, as previously described.^41^^,^^42^ Briefly, individual muscle fibers were segmented using an automatic threshold for laminin, and microdystrophin-positive pixels were identified using an automatic threshold for microdystrophin signal (Supplemental Figure S1). The automatic thresholds were calculated on the basis of the sarcoplasmic signal intensities within each image. Fibers with a ≥30% microdystrophin-positive perimeter were considered overall positive for microdystrophin and used to calculate the percentage microdystrophin-positive fibers relative to the total fibers for each tissue section. Fiber microdystrophin intensity was measured as the mean fluorescence intensity of all of the pixels in the microdystrophin channel within a 3-μm–wide loop following the laminin boundary of each muscle fiber. The mean microdystrophin intensity for each section was then calculated as the average of individual fiber intensities. Last, fiber size was measured as the minimum Feret diameter of each laminin-defined muscle fiber, and the average fiber size was calculated for each sample.

### Western Blot Analysis

Western blot analysis was performed, as previously described,^43^ to assess the protein content that may influence mitochondrial homeostasis. Frozen sections (8 μm thick) of quadriceps tissue were homogenized with radioimmunoprecipitation assay lysis buffer (MilliporeSigma; 20-188) that contained cOmplete mini protease inhibitor cocktail tablets (Roche, Basel, Switzerland; number 11836153001) and PhosSTOP EASYpack phosphatase inhibitor cocktail tablets (Roche; number 04906837001). Criterion TGX Stain-Free gels (Bio-Rad, Hercules, CA; catalog number 5678094) were loaded with the protein isolates and run for 30 minutes at 80 V, followed by 1.5 hours at 120 V. Gels were activated for stain-free protein expression using the Bio-Rad ChemiDoc MP system. After transfer, polyvinylidene difluoride membranes were imaged for total protein expression. Antibodies against latent TGF-β–binding protein 4 (LTBP4; Novus Biologicals, Centennial, CO; NBP2-43671; 1:500), TGF-β (Novus Biologicals; NBP2-46108; 1:2000), annexin A6 (ANXA6; ProteinTech, Rosemont, IL; 68086-1-Ig; 1:2000), mitochondrial cytochrome c oxidase subunit 2 (MTCO2; Thermo Fisher; A-6404; 1:500), SMAD4 (ProteinTech; 10231-1-AP; 1:100), dynamin-related protein 1 (DRP1; ProteinTech; 12957-1-AP; 1:1000), and Total OXPHOS Rodent WB Antibody Cocktail (Abcam; ab110413; 1:25,000) were used. Blocking and antibody dilutions were performed in 5% bovine serum albumin in tris-buffered saline with Tween-20 for LTBP4, TGF-β, ANXA6, MTCO2, DRP1, and OXPHOS. SMAD4 blocking and antibodies were performed using 2.5% milk. Secondary antibodies used were both obtained from Jackson ImmunoResearch Laboratories (West Grove, PA) against mouse (Peroxidase AffiniPure Donkey Anti-Mouse IgG; 715-035-150; 1:10,000) and rabbit (Peroxidase AffiniPure Donkey Anti-Rabbit IgG; 711-035-152; 1:5000). Enhanced chemiluminescence (MilliporeSigma; GERPN2236) was used to visualize protein and quantified using Image Lab Software version 6.1 (Bio-Rad). Protein expression was normalized to the total protein seen on the polyvinylidene difluoride Stain-Free Blot as both vinculin and β-tubulin showed differences in protein expression between genotypes.

### Dosing with AAV Microdystrophin

Male mice were treated with AAV microdystrophin (rAAV9-CK8-μDys5; Solid Biosciences) to assess the impact of microdystrophin restoration on the mitochondrial abnormalities observed in untreated mice. The mice were purchased from Jackson Laboratories at 5 weeks of age and allowed to acclimate for 1 week before dosing. At 6 weeks of life, B10-*mdx* mice were given one tail vein injection with AAV at a dose of 2.00E+14 vg/kg (*n* = 5). D2-*mdx* mice were given one tail vein injection with AAV at a dose half that of the B10-mdx mice (1.00E+14 vg/kg; *n* = 3) because of limitation in AAV volume. B10-WT (*n* = 5), B10-*mdx* (*n* = 5), D2-WT (*n* = 3), and D2-*mdx* (*n* = 3) mice received single tail vein injections of phosphate-buffered saline [vehicle (Veh)]. Of the three D2-*mdx* mice that were injected, two mice had suboptimal injections where they did not receive the full volume via tail vein, but the remaining volume was injected into the tail subcutaneously. Mice were weighed weekly during treatment and euthanized 4 weeks after injections for tissue collection and mitochondrial isolation. RCI and ΔΨ_m_ were performed on mitochondrial isolates. As described above in the Materials and Methods, one triceps muscle from each animal was used for ATP content, and one quadriceps muscle was used for histologic staining.

### Statistical Analysis

Prism 9.1.2 software (GraphPad, Inc., La Jolla, CA) was used for all statistical analysis. Outlier analysis was performed on all data sets, and outliers were removed from further evaluation. Two-way analyses of variance were used for all comparisons between the four genotypes of mice and comparisons of the SGT-001–treated mice with a Tukey multiple comparison post test. Data are presented as the means ± SEM, with significance set to *P* < 0.05 for all experiments.

## Results

### Histologic Evaluation of Mitochondrial Localization

**Figure 1:**
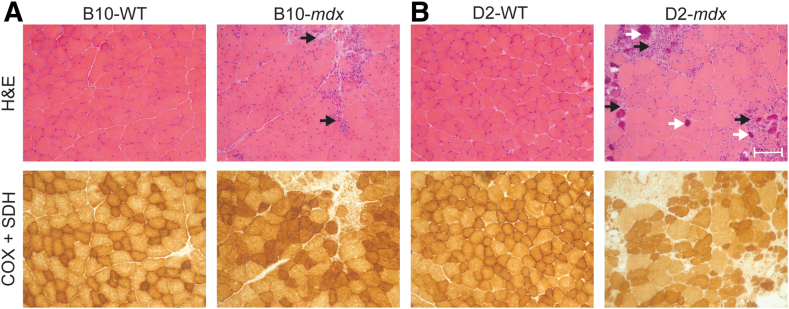
Quadriceps muscle pathology seen in Duchenne muscular dystrophy (DMD) mice. The B10-*mdx* (**A**) and D2-*mdx* (**B**) show areas of inflammation, degeneration, and regeneration that is typical in DMD pathology (**black arrows**). Additionally, D2-*mdx* animals show increased calcifications in the quadriceps (**white arrows**). The cytochrome *c* oxidase (COX) + succinate dehydrogenase (SDH) stain shows normal mitochondrial distribution in *mdx* animals aside from necrotic areas. Scale bar = 100 μm (**A** and **B**). H&E, hematoxylin and eosin.

**Supplemental Figure S2:**
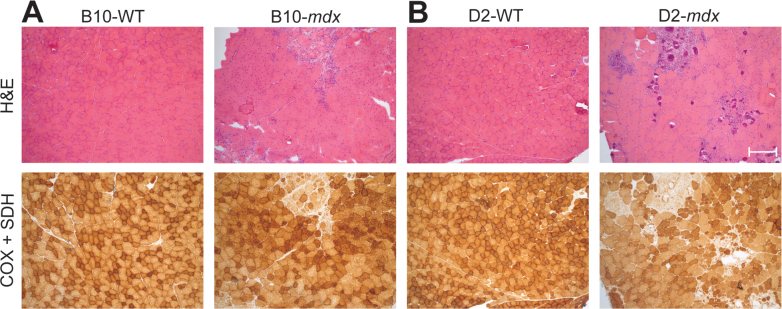
Duchenne muscular dystrophy (DMD) mice show muscle pathology at 10 weeks. Lower-magnification images showing overall muscle pathology [hematoxylin and eosin (H&E)] and mitochondrial distribution [cytochrome *c* oxidase (COX)]. **A:** The B10-*mdx* mouse exhibits DMD pathology compared with the B10-WT. **B:** The D2-*mdx* shows more severe muscle pathology than the B10-*mdx*. Oxidative fibers (dark brown) show a normal distribution in areas of *mdx* muscle not currently undergoing degeneration or regeneration. Scale bar = 200 μm (**A** and **B**). Original magnification, ×100 (**A** and **B**). SDH, succinate dehydrogenase.

Both the B10-*mdx* and D2-*mdx* mice displayed DMD pathology on H&E staining of quadriceps muscles that included decreased fiber sizes, inflammation, necrosis, and a high number of regenerating fibers compared with their respective WT counterparts (Figure 1, A and B; lower-magnification images to showcase fiber distribution provided in Supplemental Figure S2). Unlike the B10-*mdx* mice, the D2-*mdx* mice also showed numerous calcifications throughout the muscle, as previously reported.^44^ Although reductions in the density of COX and SDH staining have been previously reported in the tibialis anterior of the B10-*mdx* at 11 week of life,^16^ evaluations at 10 weeks old did not show any clear differences in COX and SDH staining patterns in quadricep muscles compared with WT animals. Both DMD mouse models showed regions devoid of mitochondrial staining in areas of active degeneration (Figure 1).

### Assessment of Mitochondrial Function

**Figure 2:**
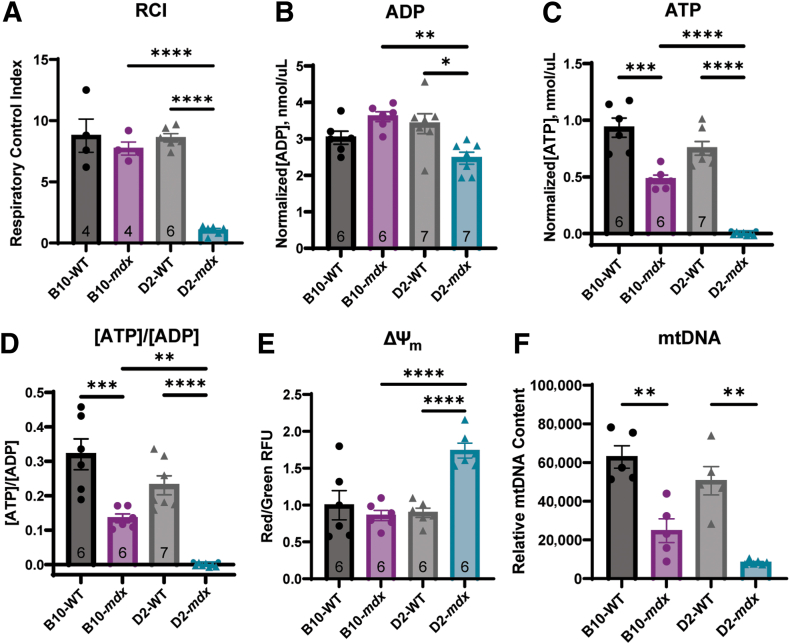
Both *mdx* mouse models exhibit unique patterns of mitochondrial dysfunction at 10 weeks of age. **A:** The D2-*mdx* shows an extreme mitochondrial respiratory deficiency, as represented by respiratory control index (RCI) values, when compared with both the B10-*mdx* and D2-WT animals. **B** and **C:** Concentrations of ADP (**B**) and ATP (**C**) were assessed in skeletal muscle and normalized to total protein in the sample. **D:** The ratio of [ATP]/[ADP] was also assessed in each animal model and showed decreased ratios in *mdx* models. **E:** Mitochondrial membrane potential (ΔΨ_m_) was evaluated by the red/green fluorescence ratio using JC-1 (5,5′,6,6′-tetrachloro1,1′,3,3′-tetraethylbenzimidazolocarbocyanine iodide) on mitochondrial isolates to establish the integrity of the mitochondrial membrane. **F:** Last, the relative amount of mitochondrial DNA (mtDNA) to nuclear DNA was assessed to evaluate overall mitochondrial number. ∗*P* < 0.05, ∗∗*P* < 0.01, ∗∗∗*P* < 0.001, and ∗∗∗∗*P* < 0.0001.

Mitochondrial respirometry was used to establish overall mitochondrial function in both animal models. Glutamate, malate, and ADP were added to the sample to provide the necessary substrates for mitochondrial respiration. The mitochondria consume oxygen over time, and the changes to oxygen content in the solution were used to calculate the RCI value. The RCI is used to evaluate how well the mitochondria are respiring, with a higher oxygen-consumption-over-time ratio indicating better mitochondrial health. The B10-*mdx* mice showed no changes to RCI values when compared with B10-WT mice (Figure 2A). However, D2-*mdx* mice showed no response to the addition of ADP in the respiratory chamber and therefore did not exhibit any mitochondrial respiration that could be detected by the apparatus (Figure 2A). The D2-*mdx* mice had a significant reduction in RCI compared with both the D2-WT and the B10-*mdx* mice.

To evaluate mitochondrial output, the concentrations of ATP, ADP, and ΔΨ_m_ were assessed. No significant differences were seen between the two WT strains in any evaluation. The B10-*mdx* mice had no changes in ADP content (Figure 2B), but exhibited a significant decrease in ATP (Figure 2C), leading to a significant decrease in the ratio of [ATP]/[ADP] (Figure 2D) compared with B10-WT controls. D2-*mdx* mice showed insignificantly reduced levels of ADP compared with D2-WT and B10-*mdx* mice (Figure 2B). ATP concentrations (Figure 2C) were undetectable in D2-*mdx*, causing the ratio of [ATP]/[ADP] to be 0 (Figure 2D). This was significantly lower than the levels observed in both D2-WT and B10-*mdx*. No significant differences in ΔΨ_m_ were observed between B10-*mdx* and B10-WT mice (Figure 2E). However, D2-*mdx* samples did have significantly elevated ΔΨ_m_ compared with D2-WT and B10-*mdx* (Figure 2E).

Relative mtDNA content was measured in both mouse models of DMD and their respective WT control strains using quadricep muscles (Figure 2F). No significant differences in mtDNA content were observed between the two WT strains of mice. The B10-*mdx* mice had a significant reduction in the relative amount of mtDNA compared with the B10-WT mtDNA. The D2-*mdx* muscle also showed a significant reduction in mtDNA compared with the D2-WT mtDNA. However, there was no significant difference in the relative amount of mtDNA between the B10-*mdx* and the D2-*mdx* muscles.

**Figure 3:**
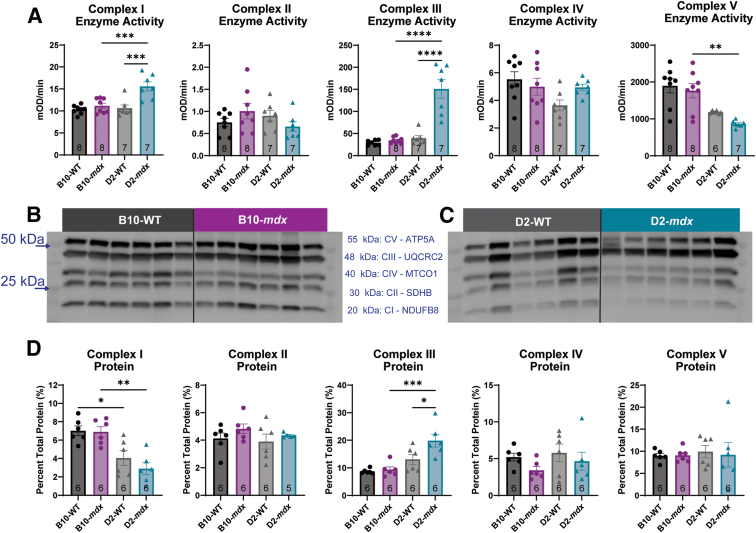
D2-*mdx* animals have differences in electron transport chain (ETC) protein expression and enzyme activity. The activity of each ETC enzyme was assessed as the change in molar optical density (mOD) over time. **A:** D2-*mdx* mice show altered enzyme activity in complexes (Cs) I, III, and V. B10-*mdx* mice show no changes to enzyme function in any ETC complex. Protein expression of subunits of each ETC enzyme were assessed in B10-*mdx*, D2-*mdx*, and WT animals. **B** and **C:** Protein expression was normalized to total protein on the blot. D2-WT and D2-*mdx* mice show a reduction in the relative protein expression of complex I compared with B10-WT and B10-*mdx* mice, respectively. **D:** D2-*mdx* mice also show an increase in relative protein expression in complex III compared with both B10-*mdx* and D2-WT mice. ∗*P* < 0.05, ∗∗*P* < 0.01, ∗∗∗*P* < 0.001, and ∗∗∗∗*P* < 0.0001. MTCO1, mitochondrial cytochrome c oxidase subunit 1; NDUFB8, NADH dehydrogenase 1 beta subcomplex subunit 8; SDHB, succinate dehydrogenase iron sulfur subunit; UQCRC2: ubiquinol-cytochrome c reductase core protein 2.

To investigate if changes in RCI values were due to altered ETC function, enzyme function assays were performed on mitochondrial isolates. Similar to RCI values, the B10-*mdx* mice showed no significant changes to ETC enzyme function in any of the ETC enzymes (Figure 3A). In contrast, the D2-*mdx* mice showed significant increases in enzyme activity in complexes I and III compared with D2-WT and B10-*mdx* mice (Figure 3A). A significant decrease in ATPase enzyme activity was seen in complex V between B10-*mdx* and D2-*mdx* mice, but no significant changes were seen within the models (Figure 3A). Although these changes of ETC activity were statistically significant, they were not sufficiently abnormal to explain the extreme deficits in RCI seen in the D2-*mdx* mice or explain why the RCI data from D2-*mdx* mice were so different from the B10-*mdx* model.

To assess for differences in ETC enzyme content between the models, relative expression levels of subunit proteins representing each complex were measured via Western blot analysis (Figure 3, B and C). There was a significant reduction in complex I [subunit 8; NADH dehydrogenase 1 beta subcomplex subunit 8 (NDUFB8)] protein expression between B10-WT and D2-WT mice and between B10-*mdx* and D2-*mdx* mice (Figure 3D). However, no significant changes were observed in complex I between WT and *mdx* of the same genetic background. No significant differences in complex II (subunit B; SDHB) levels were observed between any of the mouse lines (Figure 3D). A significant increase in complex III [subunit 2; ubiquinol-cytochrome c reductase core protein 2 (UQCRC2)] protein expression was observed between the B10-*mdx* and D2-*mdx* mice as well as between the D2-WT and D2-*mdx* mice (Figure 3D); however, no differences were noted in complex III expression between B10-WT and B10-*mdx* mice (Figure 3D). No significant differences in the levels of complex IV (subunit 1; MTCO1) (Figure 3D) or complex V (subunit α; ATP5A) (Figure 3D) levels were observed between any of the mouse lines. Overall, B10-*mdx* mice showed a relatively mild mitochondrial phenotype in skeletal muscles at 10 weeks of age, whereas the D2-*mdx* mice displayed a more severe mitochondrial phenotype with reduced RCI values, decreased ADP and ATP, altered ETC enzyme activities, and changes to complex III protein expression.

### Evaluation of D2 Genetic Modifiers

**Figure 4:**
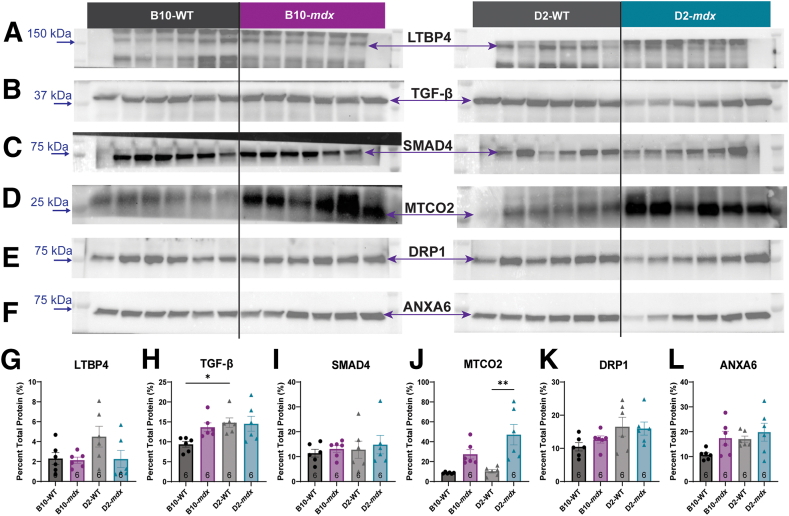
Relative protein expression changes to the latent transforming growth factor (TGF)-β–binding protein 4 (LTBP4) pathway and annexin A6 (ANXA6). LTBP4 is a known genetic modifier in human patients with Duchenne muscular dystrophy and impacts TGF-β signaling. TGF-β acts on SMAD4, which directly interacts with mitochondrial cytochrome c oxidase subunit 2 (MTCO2) and can cause mitochondrial fragmentation via dynamin-related protein 1 (DRP1). **A**–**L:** All proteins were assessed for expression levels (**A**–**F**) relative to total protein (**G**–**L**). **H:** TGF-β levels were elevated in D2-WT mice compared with B10-WT mice, and complex IV subunit 2 (MTCO2) was elevated in the D2-*mdx* mice compared with D2-WT. ∗*P* < 0.05, ∗∗*P* < 0.01.

The D2-*mdx* mouse has a more severe mitochondrial deficiency than the B10-*mdx*, even though they share the same dystrophin gene mutation. However, both the D2-WT and the D2-*mdx* mice have a 12–amino acid deletion in LTBP4 (a known modifier of DMD) that is thought to influence SMAD4 signaling via TGF- β.^44^^,^^45^ Western blot analyses were performed to assess expression levels of proteins involved in the LTBP4 pathway (Figure 4, A–E and G–K). LTBP4 is required for the sequestration of TGF-β in its latent form, and mutations in LTBP4 that impact this function have been shown to alter TGF-β pathway activity.^46^^,^^47^ TGF-β acts on SMAD4, which directly interacts with complex IV subunit 2 (MTCO2), altering DRP1 signaling, and subsequently mitochondrial fission. No significant differences in total LTBP4 protein levels were seen between any of the four mouse strains (Figure 4, A and G). TGF-β showed significant changes between the D2-WT and B10-WT mice (Figure 4, B and H). However, there were no significant changes to SMAD4 protein expression (Figure 4, C and I). Subunit 2 of complex IV (MTCO2) had a significant increase in protein expression between the D2-WT and D2-*mdx* mice (Figure 4, D and J) and a trend toward significance in the B10-WT versus B10-*mdx* mice (Figure 4, D and J). As with the ETC enzymes, the protein expression patterns of these genetic modifiers do not fully explain the mitochondrial phenotype seen in the D2-*mdx* model.

Both the D2-WT and the D2-*mdx* mice also have a dysfunctional *Anxa6* gene.^44^ ANXA6 is another known modifier of DMD^48^^,^^49^ and has been shown to interact with DRP1 to regulate mitochondrial fragmentation.^50^ When protein expression levels were assessed, no significant differences were observed in relative DRP1 (Figure 4, E and K) or ANXA6 expression between groups (Figure 4, F and L). With such minimal differences in the expression levels of proteins in the LTBP4 pathway or ANXA6, it was unlikely that the more severe mitochondrial phenotype in the D2-*mdx* mice was caused by the presence of these DMD modifiers.

### Effects of AAV Microdystrophin Gene Therapy on Mitochondrial Function

**Figure 5:**
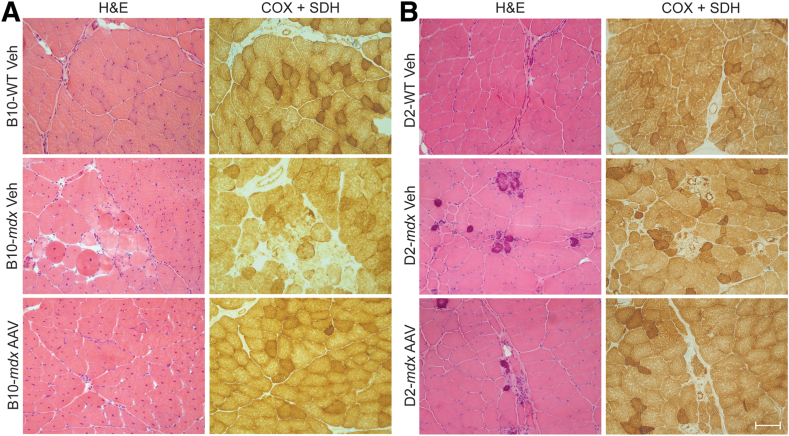
Dosing with adeno-associated virus (AAV) SGT-001 improved Duchenne muscular dystrophy (DMD) pathology in both B10-*mdx* and D2-*mdx* mice. **A:** The B10-*mdx* AAV mice received a 2.00E+14 vg/kg dose of AAV and show significant improvement in DMD pathology 4 weeks later. **B:** The D2-*mdx* mice received a 1.00E+14 vg/kg dose of AAV and show significant improvement, but not complete reversal, of DMD pathology after 4 weeks of treatment. Scale bar = 100 μm (**A** and **B**). COX, cytochrome *c* oxidase; H&E, hematoxylin and eosin; SDH, succinate dehydrogenase; Veh, vehicle.

**Figure 6:**
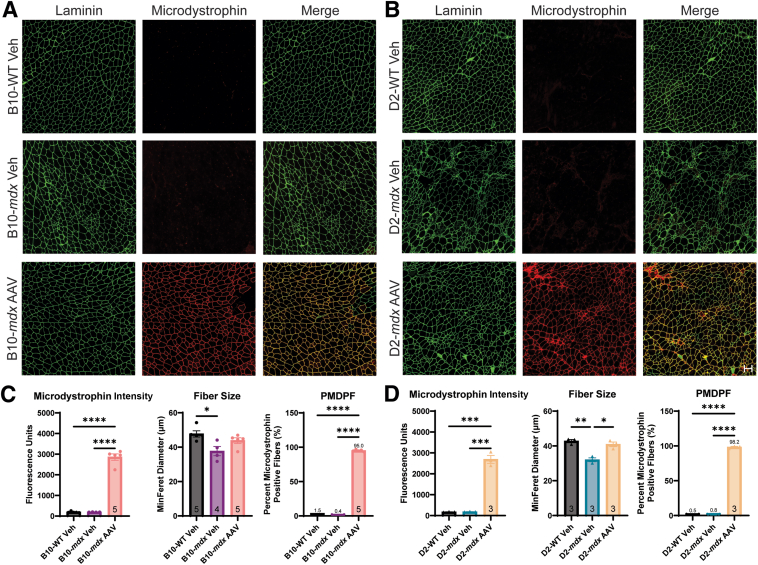
Microdystrophin expression in *mdx* mice. All mice were stained for laminin expression to identify muscle fibers. **A** and **B:** The B10-WT vehicle (Veh), B10-*mdx* Veh (**A**), D2-WT Veh, and D2-*mdx* Veh (**B**) show no microdystrophin expression. **A**–**D:** B10-*mdx* adeno-associated virus (AAV; **A**) and D2-*mdx* AAV (**B**) treated mice show robust microdystrophin expression (**C** and **D**). **C** and **D:** Fiber sizes were improved in both AAV-treated groups. The percentage microdystrophin-positive fibers (PMDPF) was >95% in the B10-*mdx* mice and >98% in the D2-*mdx* mice. ∗*P* < 0.05, ∗∗*P* < 0.01, ∗∗∗*P* < 0.001, and ∗∗∗∗*P* < 0.0001. Scale bar = 100 μm (**A** and **B**). Original magnification, ×200 (**A** and **B**).

**Supplemental Figure S3:**
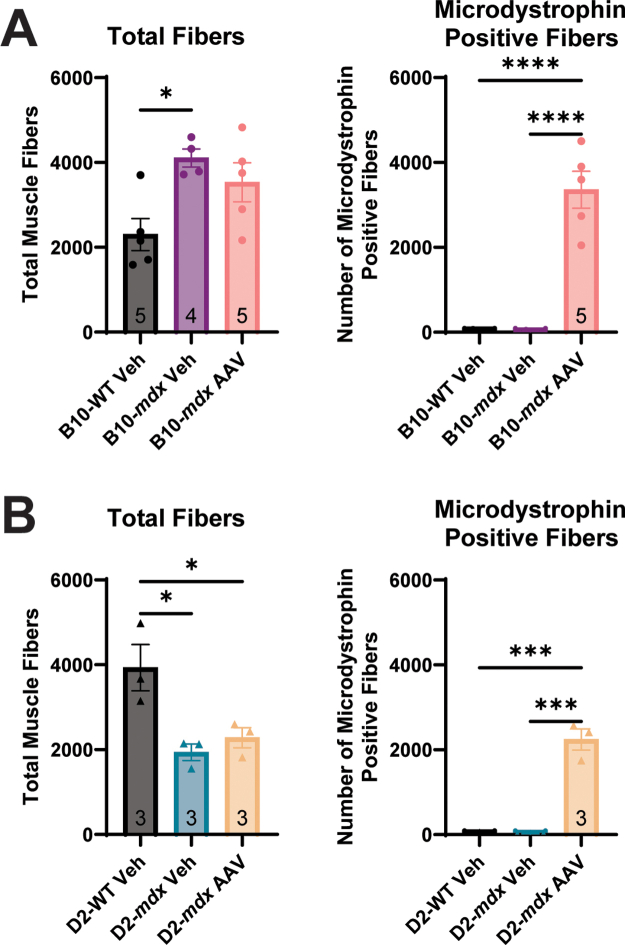
Muscle fiber quantification. B10-*mdx* mice have an increase in the total number of muscle fibers present. **A:** B10-*mdx* adeno-associated virus (AAV) mice had muscle fibers positive for microdystrophin and had overall fewer total fibers. D2-*mdx* vehicle (Veh) mice had fewer muscle fibers than D2-WT Veh mice. **B:** Although treatment with AAV leads to a significant number of fibers expressing microdystrophin, there was no attenuation to WT numbers of fibers. These quantifications were used to calculate the percentage microdystrophin-positive fibers. ∗*P* < 0.05, ∗∗∗*P* < 0.001, and ∗∗∗∗*P* < 0.0001.

Mice were systemically administered microdystrophin AAV (rAAV9-CK8-μDys5) via the tail vein to assess how well this therapeutic approach could improve the mitochondrial dysfunction observed in these models. Treated mice from both strains showed improvements in DMD pathology based on H&E staining (Figure 5), and COX + SDH staining showed no dramatic differences in mitochondrial localization in the AAV groups compared with vehicle controls (Figure 5). Immunofluorescence staining for laminin showed expression in both WT and *mdx* myofibers and was used to quantify the total number of muscle fibers present (Supplemental Figure S3, A and B). AAV-treated groups displayed an increase in microdystrophin expression based on immunofluorescence staining using MANDYS106, which is specific for therapeutic microdystrophin (Figure 6, A and B). Microdystrophin expression showed high staining intensity in AAV-treated mice (Figure 6, C and D) and was quantified (Supplemental Figure S3, A and B). B10-*mdx* muscle fiber size was significantly smaller than B10-WT fibers and was attenuated with AAV treatment (Figure 6C). The size of muscle fibers was significantly larger in the AAV-treated D2-*mdx* mice compared with D2-*mdx* Veh-treated mice and was no longer significantly different than D2-WT fibers (Figure 6D). Although the D2-*mdx* mice received a lower concentration of AAV, both mouse models expressed >95% microdystrophin-positive fibers (Figure 6, C and D).

**Figure 7:**
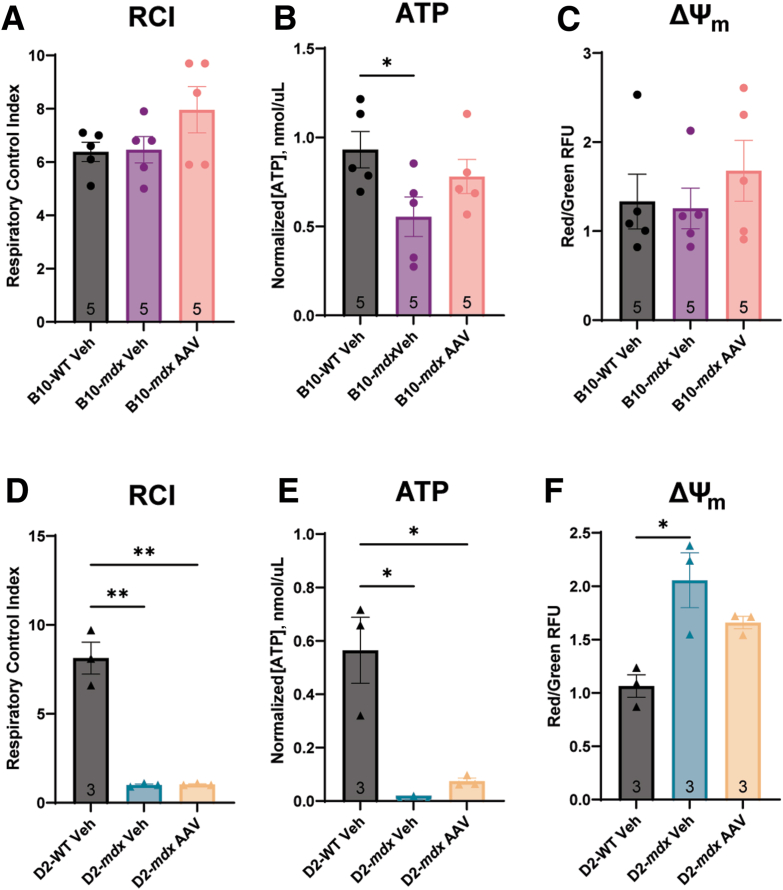
Treatment with SGT-001 adeno-associated virus (AAV) produces modest improvements to mitochondrial physiology. Respiratory control index (RCI), ATP content, and mitochondrial membrane potential (ΔΨ_m_) were assessed in AAV treatment of the B10-*mdx* (**A**–**C**, respectively) and D2-*mdx* mice (**D**–**F**, respectively) to assess mitochondrial function when dystrophin is restored. B10-*mdx* ATP levels were attenuated to WT levels; however, similar improvements were not seen in the D2-*mdx* mice. The D2-*mdx* did have a reduction to ΔΨ_m_ so that it was no longer significantly elevated compared with D2-WT mice. ∗*P* < 0.05, ∗∗*P* < 0.01. RFU, relative fluorescence unit; Veh, vehicle.

Only RCI, ATP, and ΔΨ_m_ were evaluated in the AAV-treated groups as these were all significantly different between untreated *mdx* mice and their WT counterparts, as described above in the Materials and Methods. There were no significant differences in RCI and ΔΨ_m_ in B10-*mdx* untreated or AAV-treated mice compared with B10-WT mice (Figure 7, A and C). Although the vehicle-treated B10-*mdx* mice maintained a deficit in ATP amounts, AAV-microdystrophin treatment normalized ATP levels (Figure 7B) to that of WT animals, indicating a restoration of ATP production with microdystrophin replacement.

However, the AAV-microdystrophin–treated D2-*mdx* mice showed no improvement in RCI values (Figure 7D) or ATP content (Figure 7E) compared with D2-*mdx* vehicle only controls. The AAV-microdystrophin–treated D2-*mdx* animals did show decreased ΔΨ_m_ compared with vehicle-treated controls and were no longer significantly different from D2-WT mice (Figure 7F), thereby restoring normal membrane potential.

**Figure 8:**
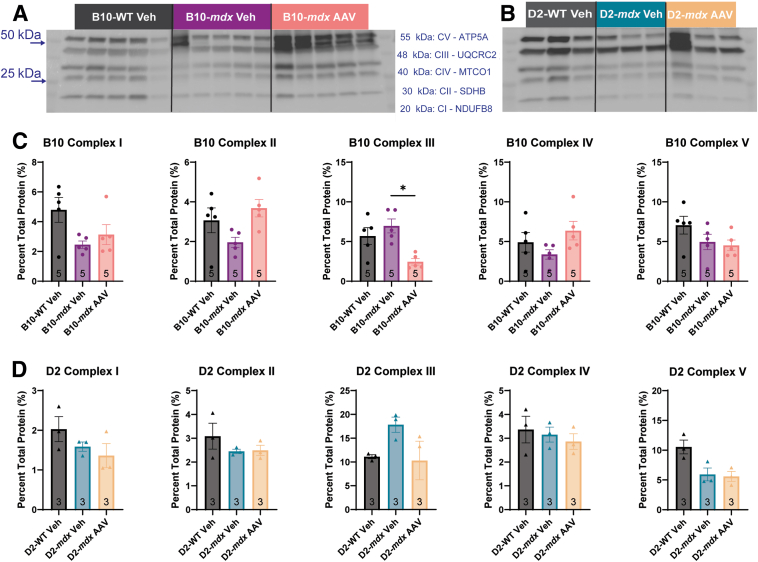
Relative protein expression of electron transport chain (ETC) enzymes in adeno-associated virus (AAV)–treated mice. Abcam's Western Blot Antibody Cocktail was used to assess expression of a subunit from each enzyme. Blots for each genotype are shown (**A** and **B**) with protein expression shown relative to total protein (**C** and **D**). AAV-treated B10-*mdx* mice show a significant reduction in complex (C) III protein expression (**C**), whereas D2-*mdx* vehicle and AAV-treated mice show no significant changes to any ETC protein expression (**D**). ∗*P* < 0.05. Veh, vehicle.

There were no significant differences observed in ETC enzyme protein expression levels between B10-WT and B10-*mdx* Veh control groups. The AAV-microdystrophin–treated B10-*mdx* mice did show significantly reduced complex III expression levels compared with both B10-WT and B10-*mdx* vehicle-treated groups (Figure 8, A–C). For the D2-*mdx* mice, no significant changes to ETC enzyme protein expression were seen (Figure 8D).

**Supplementary Figure S4:**
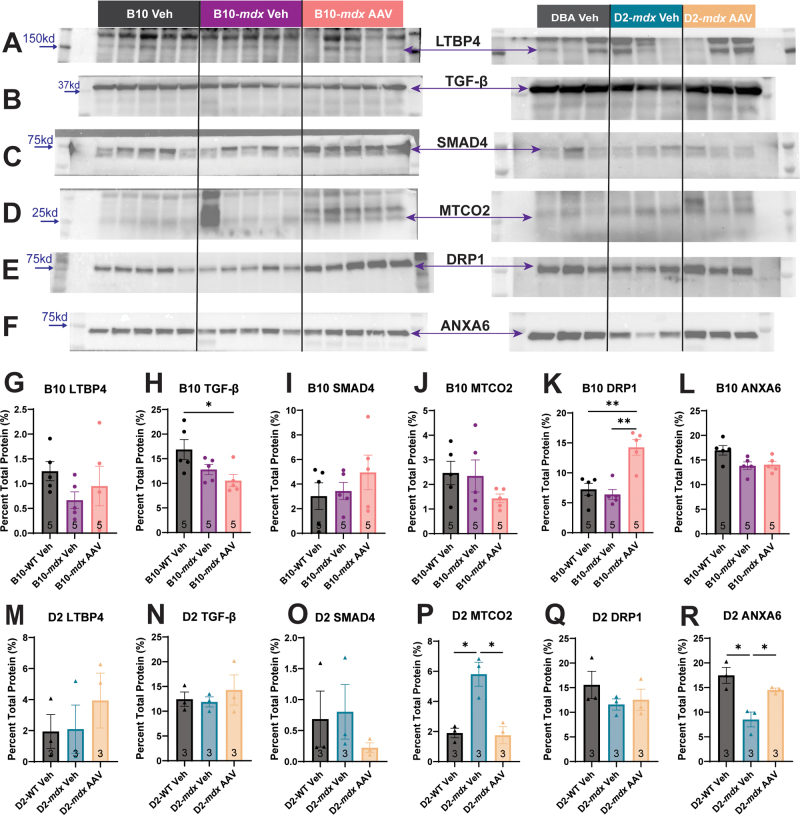
Assessment of the latent transforming growth factor (TGF)-β–binding protein 4 (LTBP4) pathway and annexin A6 (ANXA6) with adeno-associated virus (AAV) treatment. **A**–**F:** Blots for each protein and genotype are shown. **G**–**R:** Protein expression was analyzed using total protein and relative expression levels in vehicle (Veh)– and AAV-treated B10-*mdx*, D2-*mdx*, and their WT counterparts, as shown. B10-*mdx* AAV-treated mice show a reduction in TGF-β expression and an increase in dynamin-related protein 1 (DRP1) protein expression. D2-*mdx* AAV-treated mice show a reduction in mitochondrial cytochrome c oxidase subunit 2 (MTCO2) and an increase in ANXA6 relative protein levels, thereby making them similar to WT. ∗*P* < 0.05, ∗∗*P* < 0.01.

To evaluate whether AAV-microdystrophin treatment impacted the LTBP4 pathway, ANXA6, or DRP1 levels, Western blot analyses were performed, as described above in the Materials and Methods. No changes in LTBP4, SMAD4, MTCO2, or ANXA6 were observed across any of the B10 groups (B10-WT Veh, B10-*mdx* Veh, or B10-*mdx* AAV) (Supplemental Figure S4). B10-*mdx* AAV-treated mice did show a significant reduction in TGF-β compared with B10-WT Veh-treated mice (Supplemental Figure S4). Treatment with AAV also significantly increased the protein expression levels of DRP1 in AAV-treated animals compared with both B10-*mdx* Veh and B10-WT Veh mice (Supplemental Figure S4).

No changes in LTBP4, TGF-β, SMAD4, or DRP1 were observed across any of the D2 groups (D2-WT Veh, D2-*mdx* Veh, or D2-*mdx* AAV) (Supplemental Figure S4). AAV-microdystrophin treatment of the D2-*mdx* mice significantly reduced MTCO2 levels back to that of D2-WT mice and significantly increased ANXA6 levels back to D2-WT amounts (Supplemental Figure S4). Overall, this AAV-microdystrophin treatment strategy had only a minimal effect on mitochondrial phenotypes and protein expression levels in these *mdx* models.

## Discussion

DMD affects approximately 1 in 5000^1^ males, and extensive research has been performed on the implications of dystrophin deficiency in muscle tissue in animal models. Two of the most frequently used mouse models (B10-*mdx* and D2-*mdx*) are often viewed as interchangeable models of DMD, and comprehensive life histories of both animal models have been previously reported.^44^^,^^51^ The B10-*mdx* mouse has historically been the most widely used mouse model of DMD, but has a milder phenotype than human patients,^52^, ^53^, ^54^ whereas the D2-*mdx* mice show a more severe pathology that progressively worsens with age, making the model more comparable to human patients.^44^^,^^51^ The B10-*mdx* skeletal muscle pathology presents at lower levels than that in human patients and plateaus at approximately 3 to 4 months of age.^44^^,^^52^ Both mouse models have elevated pyruvate kinase and creatine kinase, small muscle fibers, centralized nuclei, reduced dystrophin expression, necrosis, and inflammation in skeletal muscles,^5^ but D2-*mdx* mice also have lower regenerative capacities, elevated fibrosis, fat deposition, and calcification.^7^^,^^44^^,^^45^

Although many phenotypes have been evaluated in the B10-*mdx* and D2-*mdx* models, metabolic changes in both DMD animal models have yet to be thoroughly investigated at a common time point using the same methods. For these studies, 10-week–old mice were used, as that time point has previously shown differences, including increased calcification, necrosis, fibrosis, regeneration, and inflammation, between the D2-*mdx* and B10-*mdx*.^44^^,^^51^ The B10-*mdx* mouse showed abnormalities in mitochondrial biology that precede DMD muscle pathology.^16^ Beginning at the onset of myofiber damage in the B10-*mdx*, there are changes in gene expression and proteins associated with mitochondrial dynamics, decreases in mtDNA,^16^ COX and SDH staining,^16^ mitochondrial RCI,^16^, ^17^, ^18^^,^^21^, ^22^, ^23^, ^24^, ^25^ ETC complex activity,^16^^,^^18^, ^19^, ^20^, ^21^, ^22^ ATP content,^22^^,^^26^^,^^27^ and altered mitochondrial anatomy and localization on electron microscopy.^16^^,^^28^^,^^29^ Alterations in mitochondrial function have also been reported in D2-*mdx* mice at 4 weeks of age. These changes include decreases in respiratory capacity, decreases in complex III protein levels, elevated hydrogen peroxide, and a reduction in calcium retention capacity of the mitochondria.^31^^,^^55^^,^^56^

The laboratory previously established a series of assessments to evaluate overall mitochondrial health in NM.^14^^,^^15^ NM affects approximately 1 in 50,000 children^11^ and varies widely in both genetic causation and phenotypic severity.^12^^,^^57^ One of the main contributors to NM pathology is improper filament construction and subsequent sarcomeric dysfunction.^58^, ^59^, ^60^ Those studies identified a mitochondrial phenotype that correlates with disease severity. The severely affected nebulin conditional knockout mouse showed the most severe mitochondrial phenotype, including mitochondrial mislocalization, severely decreased mitochondrial respiration, undetectable ATP concentrations, and increased membrane potential.^14^ However, the minimally affected Tg*ACTA1*^D286G^ had only minor changes to mitochondrial location and no significant functional changes compared with WT control mice.^15^ In nebulin-deficient NM, myosin fibers do not enter into a super relaxed state, thereby increasing ATP consumption in resting muscle fibers,^61^ which may account for the decrease in ATP observed in the previous work.^14^

Here, the same methods were used to evaluate the mitochondrial function of the B10-*mdx* and D2-*mdx* at a common time point. It has been previously reported that D2-*mdx* mice display a more severe DMD pathology compared with the B10-*mdx* mice at 10 weeks of life.^44^ H&E staining revealed increased degeneration and regeneration, fibrosis, fatty infiltration, and calcifications in the D2-*mdx* mice in comparison to B10-*mdx* mice,^1^^,^^44^ which has been previously linked to the genetic modifiers in the D2-WT background.^9^ Both COX and SDH staining work by oxidizing a dye in solution based on enzyme activity and subsequently are used as a qualitative measure of enzyme function and mitochondrial location.^62^^,^^63^ Decreased muscle fibers displaying a high density of COX or SDH staining have been previously reported in the tibialis anterior of 11-week–old B10-*mdx*^16^ mice. At 10 weeks, B10-*mdx* and D2-*mdx* quadriceps had COX + SDH staining patterns similar to WT mice in areas of muscle that were not undergoing active remodeling and necrosis. Areas of myonecrosis displayed decreased mitochondrial staining using the COX + SDH stain, but this is appropriate for degenerating tissue as both stains produce a color change based on enzyme activity. Although the D2-*mdx* mouse does show more DMD pathology, the modest increase in pathologic severity does not fully explain the severe mitochondrial insufficiency seen in the D2-*mdx* mice.

With respect to mitochondrial function, D2-*mdx* mice exhibit a much more severe mitochondrial deficit than B10-*mdx* mice.^31^ Mitochondrial isolates from the whole muscle tissue of the D2-*mdx* mouse had no respiratory response to glutamate/malate and ADP, therefore presenting as an RCI value of 1, the slope of a straight line. The RCI is normalized using the total protein present in the sample and not strictly to mitochondrial proteins. It has been shown that D2-*mdx* mice have a reduction in the number of mitochondria present in skeletal muscle using electron microscopy^31^; therefore, it is possible that each crude mitochondrial sample of D2-*mdx* tissue, while having comparable total protein concentrations, had fewer mitochondria present than WT or B10-*mdx* samples, thereby limiting the respiratory capacity of the sample.

The function of each enzyme of the ETC was assessed to evaluate if enzymatic dysfunction was responsible for the reduction in RCI. It has been previously speculated that B10-*mdx* mice have an impairment in complex I that leads to decreases in complex I–driven mitochondrial respiration^19^, ^20^, ^21^; however, no changes in complex I activity in the B10-*mdx* mice were observed at this age. It has also been previously reported that the D2-*mdx* have a decrease in complex I and II stimulated respiration at 4 weeks of age, but individual enzyme activities were not assessed in those studies.^55^ D2-*mdx* mice did have elevated complex I activity compared with B10-*mdx* and D2-WT mice, but neither *mdx* mouse strain showed differences in complex II or complex IV enzyme activity with individual enzyme assays. This was further validated by the normal staining patterns on SDH + COX in *mdx* mouse samples not undergoing degeneration and regeneration. Although there was an increase in complex I and III individual enzyme activity, only complex III showed an increase in relative protein expression in the D2-*mdx* mice compared with both the B10-*mdx* and the D2-WT. Complex V also showed a decrease in its ATPase activity in both D2-WT and D2-*mdx* compared with B10-WT and B10-*mdx*, respectively. These minor changes to ETC activity and protein expression still do not seem to fully explain the RCI phenotype.

Interestingly, even with unaltered enzyme function and protein expression, the B10-*mdx* mice did display alterations in ADP and ATP content. The B10-*mdx* mice have an increase in ADP, decreases in both ATP and the ratio of [ATP]/[ADP], indicating a shift in metabolism toward glycolysis,^64^ but no change in the ΔΨ_m_. In contrast, the D2-*mdx* mice exhibit decreases in the concentrations of ADP, ATP, and the [ATP]/[ADP] ratio, and a significant increase in ΔΨ_m_. It is possible that the observed increase in ΔΨ_m_ in the D2-*mdx* is due to the altered ETC activity, that ATP is being consumed faster than it can be produced, or complex V is unable to respond to increased ADP levels, as evidenced by the complete lack of RCI values. The assay used here assessed complex V's ability to pump hydrogen back across the mitochondrial membrane, and is not indicative of complex V's ability to produce ATP, and therefore, needs to be further investigated.

Although *mdx* muscle is not as severely disorganized as NM muscle, *mdx* mice have shown changes to actin and myosin protein content^65^ as well as an increase in cleaved actin.^28^ This, combined with suboptimal rates of energy conversion,^22^^,^^66^ may be contributing to the decrease in ATP content^22^ seen here in *mdx* mice. Subsequently, muscles cannot generate an adequate amount of ATP to keep up with cellular demand and, thus, can only maintain suboptimal ATP levels that are not sufficient for long-term function.^22^ There may also be a threshold of cellular or mitochondrial impairment that needs to be reached before these deficits become consequential, where more severe disease phenotypes (such as nebulin conditional knockout and D2-*mdx*) show more mitochondrial dysfunction compared with less severe disease phenotypes (Tg*ACTA1*^D286G^ and B10-*mdx*).

The D2-*mdx* mice show a decrease in relative mtDNA content compared with both WT strains as well as the B10-*mdx*, which implies that a certain amount of healthy mtDNA is needed to maintain mitochondrial function in DMD.^67^ Although most mitochondrial proteins are encoded in the nuclear DNA, the mtDNA plays a critical role in mitochondrial health. Even in healthy populations, the accumulation of mtDNA mutations or decreased mtDNA is associated with disease states.^68^ The B10-*mdx* mice have approximately 40% of the B10-WT mtDNA, whereas, D2-*mdx* muscle contains <20% of D2-WT mtDNA. This may indicate that there is a critical amount of mtDNA needed for healthy mitochondrial function, and below that threshold, mitochondria cannot maintain proper function and ATP levels. This is supported by the observation that B10-*mdx* muscle has approximately twice as much mtDNA as D2-*mdx* muscle and exhibits fewer mitochondrial deficiencies and better mitochondrial function overall.

The lack of mitochondrial respiration (RCI values) in the D2-*mdx* and alterations in ATP content are likely due to a combination of factors, including altered ETC enzyme protein and activity, severely reduced mtDNA, increased ΔΨ_m_, alterations in fiber type proportions, and potentially the sensitivity of the equipment. Crude mitochondrial samples were used in all assays so other cellular proteins may have been contributing to the total protein in the sample. It is speculated that only approximately 20% to 40% of mitochondria are isolated from skeletal muscle after homogenization.^69^ Because all assays were performed on the basis of total protein, the sample may not have appropriately represented only mitochondria as other cellular proteins were still present. Therefore, although the same amount of protein was added to each assay, different amounts of functional mitochondria may have been added to each assay. It is also possible that the equipment used was not sensitive enough to establish RCI values on the D2-*mdx* mice as it could not detect any changes in oxygen content of the sample. Last, it was speculated there may be variable changes between specific muscle groups based on relative abundance of fast- and slow-twitch fibers that are masked when several muscle tissues are pooled together. Increases in type IIA fiber content have been reported in the D2-*mdx* mouse^56^; however, no differences in the maximal respiratory capacity have been found between mitochondria isolated from fast- or slow-twitch fibers,^69^ therefore making it unlikely to change results if only fast or slow fibers were examined. Even though both the B10-*mdx* and D2-*mdx* mice are models of the same disease, as with previous descriptions of overall severity in these models, the D2-*mdx* mice show a much more severe mitochondrial phenotype than the B10-*mdx* mice.

It was hypothesized that differences between the B10-*mdx* and D2-*mdx* may also be attributed to genetic modifiers acting on secondary cellular processes that may be responsible for the decreases in regenerative capabilities,^1^^,^^44^ increased calcified muscle fibers,^44^ and alterations to mitochondrial function. Differences in DMD severity have been linked to genetic modifiers in the human population. Two of these modifiers also exist in the D2-*mdx* mouse: *Ltbp4*^8^ and *Anxa6*.^9^ Several haplotypes of *LTBP4* in humans have been reported to alter DMD severity and slow disease progression.^8^^,^^46^ LTBP4 is required for maintaining the proper secretion, storage, and folding of TGF-β.^70^ Insertions or deletions into the protein have been shown to alter proteolysis^8^ and LTBP4's ability to sequester TGF-β.^47^ The *Ltbp4* polymorphism seen in the D2-*mdx* mice is a deletion of 12 amino acids that increases proteolysis and alters LTBP4's ability to bind latent TGF-β,^8^ which increases TGF-β bioavailability and SMAD signaling.^8^ Increases in active TGF-β have been linked to the accumulation of fibrosis and calcification and decreases in myogenesis in the D2-*mdx* mouse.^71^ Additionally, muscle damage induces SMAD signaling, and elevated levels of phosphorylated SMAD2/3 have been reported in muscular dystrophy.^72^ These changes in TGF-β and SMAD signaling have been associated with more severe DMD phenotypes^47^ and changes in mitochondrial biology.^8^^,^^46^^,^^47^ Once phosphorylated, SMAD2/3 complexes with SMAD4, where it then enters the nucleus to induce gene transcription.^72^ SMAD4 was chosen for analysis because not only does it bind with active SMAD2/3 and is essential for SMAD signaling, it also specifically interacts with cytochrome c oxidase subunit 2 (MTCO2), which is involved with stress-induced apoptosis, degenerative diseases,^73^ and muscle injury.^72^

The D2-WT mice have an increase in TGF-β expression compared with B10-WT mice, which is consistent with a dysfunctional LTBP4 protein. Changes in LTBP4 protein expression were not observed between the four mouse models assessed here, but the antibody used would not have been able to distinguish between the LTBP4 with and without the 12–amino acid deletion. Although it was not possible to evaluate phosphorylated SMAD2/3 here because of resource constraints, the increase in TGF-β protein expression did not correlate with an increase in the expression of SMAD4. There was an elevation in MTCO2 protein expression, the target of SMAD4, and no elevation in the protein expression of MTCO1, another subunit of complex IV, which may indicate a more active pathway even without changes in SMAD4 protein expression. Protein expression is not indicative of protein function,^74^ and changes to subunit expression of complex IV did not alter enzymatic activity as both staining patterns and the individual enzyme assays demonstrated that complex IV was not impaired. Last, DRP1 also regulates mitochondrial morphology via ANXA6,^50^ and although trends in protein expression changes were observed in ANXA6, none reached statistical significance.

To evaluate if restoring dystrophin function reversed the mitochondrial deficiencies, *mdx* mice were treated with AAV-microdystrophin (rAAV9-CK8-μDys5). Although the D2-*mdx* mice received half of the vector load (1.00E+14 vg/kg) as the B10-*mdx* mice (2.00E+14 vg/kg), both DMD genotypes expressed sufficient levels of microdystrophin to improve DMD pathology on H&E and show clear microdystrophin expression in the quadriceps via immunofluorescence. Even at half of the therapeutic dose, D2-*mdx* mice showed equivalent microdystrophin expression to the B10-*mdx* mice treated with twice the viral load. Treatment with AAV-microdystrophin caused only mild improvements to the ATP concentration in the B10-*mdx* mice and ΔΨ_m_ of the D2-*mdx* mice. ATP content in B10-*mdx* vehicle-treated animals was reduced, and AAV treatment normalized ATP levels back to that of the WT animals. Similar results were seen with the attenuation of ΔΨ_m_ in the D2-*mdx*. Vehicle-treated D2-*mdx* mice had elevated ΔΨ_m_, whereas AAV treatment reduced ΔΨ_m_, thereby making it not statistically different from D2-WT levels. There were no significant improvements in RCI or ATP values in AAV-treated D2-*mdx* mice in comparison to vehicle-treated animals, implying that mitochondrial health is still severely impaired 4 weeks after AAV treatment.

There are several factors that would have impacted the therapeutic results. One is that the mice were assessed 4 weeks following AAV-microdystrophin administration. It is likely that extending this time frame would result in more significant improvements in treated mice as it may require more time to improve organelle physiology after dystrophin replacement. Another factor is the age of treatment. Treating the mice at a younger age could potentially provide greater prevention of disease pathology. Finally, the gene therapy vector used in these studies makes use of the well-characterized AAV9 capsid.^75^ Although this is a valid option for preclinical proof-of-concept studies such as this, there are other AAV capsids being developed and evaluated that may be better options for skeletal muscle-specific AAV transduction.

Although the B10-*mdx* mouse model has been the most popular choice for DMD preclinical research, the newer D2-*mdx* model shows more phenotypic similarities to the human patient population with DMD, including reduced muscle weight, increased fat and fibrosis, as well as a reduction in myofiber numbers.^7^ It is important to note the extreme mitochondrial differences between these two animal models of DMD. Often, these mice are viewed as interchangeable models of the disease; however, when studying metabolic changes occurring in DMD, the B10-*mdx* animal should be used with caution as it does not show significant changes to mitochondrial biology at this relatively early time point aside from a change in ATP content. Although the exact cause for these functional changes between the two *mdx* models has not been identified, there is strong evidence that the D2-*mdx* animal has a severe mitochondrial phenotype that should be taken into consideration when using these mice for future studies.

As with any study, the current study had several limitations. Most notably, it is unclear how these different mitochondrial alterations compare with the human patient population. The D2-*mdx* has been said to be more comparable to the human disease^44^^,^^45^^,^^51^; however, without human tissue to compare these findings, it is difficult to assess if mitochondrial phenotypes may be indicative of disease progression. Additionally, it is possible that more fragile mitochondria, such as the D2-*mdx* mitochondria, may be destroyed in the isolation process, thereby skewing the results. Although *mdx* mitochondria were always isolated in tandem with WT mitochondria and all assays were run concurrently or alternating with WT samples to ensure that all samples were treated the same, this may not account for genotype-specific mitochondrial fragility. Because all *mdx* samples were able to be evaluated for membrane potential and overall enzyme function, it is unlikely that the mitochondria were completely destroyed in the isolation process; however, it is possible that going forward *mdx* tissue needs a gentler isolation method to ensure mitochondrial function.

Another limitation of the study was the use of pooled muscle samples versus individual muscles. Both isolated mitochondria and permeabilized fibers have been evaluated for differences in maximum respiratory capacity of fast- and slow-twitch fibers in the past, and no differences in ADP-stimulated respiration were identified.^69^ This suggests that mitochondria function similarly regardless of fiber type, therefore validating the use of pooled muscle samples. Previous studies with the NM mice also indicated a minimum amount of muscle tissue was needed during the isolation process to achieve protein concentrations that allowed for functional analysis. Although this method does not allow for the analysis of individual muscle groups, it does give an average mitochondrial function in muscle. Most muscles have a mixture of fiber types and, therefore, pooling muscle and getting an average mitochondrial function may be more physiologically relevant as no differences in respiratory capacity have been observed between fiber types.^69^ Additionally, if individual muscle groups would have been analyzed using these methods, many more mice would have been needed to achieve the necessary muscle mass for isolation as several mice would need to be pooled for analysis. Future studies should be performed on individual muscle groups or fibers to determine differences in mitochondrial function in individual muscles or fibers using newer methods, such as Ouroboros. Overall, this study further addresses overall mitochondrial health in two mouse models of DMD, but future studies need to be performed to determine the cause of mitochondrial dysfunction as well as determine if there are fiber-specific differences in function and how those differences relate to the human population.

## Disclosure Statement

M.W.L. is the founder, CEO, and owner of Diverge Translational Science Laboratory. M.W.L. is or has recently been a member of advisory boards for Solid Biosciences, Taysha Gene Therapies, and Astellas Gene Therapies (formerly Audentes Therapeutics). M.W.L. is also a consultant for Astellas Gene Therapies (formerly Audentes Therapeutics), Encoded Therapeutics, Modis Therapeutics, Lacerta Therapeutics, Dynacure, AGADA Biosciences, Affinia Therapeutics, Biomarin, Locanabio, Vertex Pharmaceuticals, Voyager Therapeutics, and Entrada Therapeutics. M.W.L. receives or recently received research support from Astellas Gene Therapies, Solid Biosciences, Kate Therapeutics, Prothelia, Ecogenome, Cure Rare Disease, Rocket Pharma, Ultragenyx, Carbon Biosciences, Locanabio, Regenxbio, Vita Therapeutics, Lexeo Therapeutics, Lovelace Biomedical, Prevail Therapeutics, Entrada Therapeutics, Denali Therapeutics, Epicrispr Bioscience, Dyne Therapeutics, NMD Pharma, Scholar Rock, and Satellos. J.P.G. is an employee and shareholder of Solid Biosciences. J.S., H.M., M.H., M.J.P., M.J.B., and T.A.V. are employees of Diverge Translational Science Laboratory.
