# Supplementary material for: The D2.B10-Dmdmdx/J Mouse Model of Duchenne Muscular Dystrophy Exhibits a Severe Mitochondrial Deficiency Not Observed in the C57BL/10ScSn-Dmdmdx/J Mouse
Source: Am J Pathol. 2025 Sep 30;196(2):532–49. doi: 10.1016/j.ajpath.2025.09.005 (PMC12881301; doi:10.1016/j.ajpath.2025.09.005)
Supplement: Supplemental Table S1 [file mmc1.docx]

**Supplementary Table 1: Mitochondrial assay details.**

| Protocol Element | Complex I | Complex II | Complex III | Complex IV | Complex V | ATP | ADP |
| --- | --- | --- | --- | --- | --- | --- | --- |
| Manufacturer (product number) | Abcam (ab109721) | Abcam (ab109908) | Abcam (ab109905) | Abcam (ab109911) | Cayman Chemical (#701000) | Abcam (ab83355) | Abcam (ab83359) |
| Plate Type | Immunocapture | Immunocapture | 96-Well | Immunocapture | ½ Volume 96-Well | 96-Well | 96-Well |
| Sample buffer | Isolation Buffer† | Isolation Buffer† | Isolation Buffer† | Isolation Buffer† | Isolation Buffer† | ATP Assay Buffer | ADP Assay Buffer |
| Detergent extraction | Yes | Yes | No | Yes | No | N/A | N/A |
| Muscle | 2 Quad, 2 Tri, 2 Ham, 2 Gast‡ | 2 Quad, 2 Tri, 2 Ham, 2 Gast‡ | 2 Quad, 2 Tri, 2 Ham, 2 Gast‡ | 2 Quad, 2 Tri, 2 Ham, 2 Gast‡ | 2 Quad, 2 Tri, 2 Ham, 2 Gast‡ | 1 Tri‡ | 1 Tri‡ |
| Homogenize | Yes | Yes | Yes | Yes | Yes | Yes | Yes |
| Initial Protein Concentration | 5.5mg/ml | 5.5mg/ml | 1.2mg/ml | 5.5mg/ml | 5.5mg/ml | Determined by Bradford Assay | |
| Final Protein Concentration | 300µg/ml | 1mg/ml | 0.09mg/ml | 25µg/ml | 25µg/200µL | Varies, 5μL sample/45μL Mix | |
| Reaction Solution | NADH, dye | Ubiquinone, Succinate, DCPIP | Succinate, Cytochrome c, KCN, Rotenone | Cytochrome c | Enzyme Mix, ATP, NADH, Rotenone | Reaction Mix: Probe, Converter, Developer Background Mix: Probe, Developer | |
| Incubation | 3h | 2h + 30m with Lipids (dark) | N/A | 3h | N/A | 30 min (dark) | |
| Wavelength | 450nm | 600nm | 550nm | 550nm | 340nm | 570nm | |
| Test Length | 30 min | 60 min | 20 min | 120 min | 3 min | Instant Read | |
| Analysis Time | 20 min | 10 min | 5 min | 7 min | 12 sec |  |  |
| Shaking | Yes | No | No | No | No | N/A | |
| Temperature | Room Temp | Room Temp | Room Temp | 30°C | 25°C | Room Temp | |

† Isolation buffer: 200 mM mannitol, 50 mM sucrose, 5 mM KH_2_PO_4_, 5 mM MOPS, 1 mM EGTA, and 0.1% BSA (pH adjusted to 7.15 with KOH)

‡ Quad = quadricep, Tri = triceps, Ham = hamstrings, Gast = gastrocnemius
